# Physical exercise barriers and needs in adults with congenital heart disease: a qualitative study

**DOI:** 10.1136/bmjopen-2025-102090

**Published:** 2025-07-05

**Authors:** Yara F Langeveld, Nienke ter Hoeve, Annemien van den Bosch, Danielle Robbers-Visser, Robert M Kauling, Jasmijn C van Groen, Madoka Sunamura, Harald T. Jorstad, Marjolein Snaterse

**Affiliations:** 1 Department of Cardiology, Amsterdam University Medical Center, Amsterdam, The Netherlands; 2 Department of Rehabilitation Medicine, Erasmus MC University Medical Center Rotterdam, Rotterdam, The Netherlands; 3 Capri Cardiac Rehabilitation, Rotterdam, The Netherlands; 4 Department of Cardiology, Erasmus MC, Rotterdam, The Netherlands; 5 Amsterdam Cardiovascular Sciences, Amsterdam, The Netherlands; 6 Department of Cardiology, Franciscus Gasthuis & Vlietland Hospital, Rotterdam, The Netherlands

**Keywords:** Congenital heart disease, Exercise, Patient-Centred Care, QUALITATIVE RESEARCH

## Abstract

**Abstract:**

**Objective:**

Regular physical exercise has well-known health benefits and is generally considered safe for adults with congenital heart disease (ACHD). However, many individuals with ACHD remain insufficiently physically active. This study explored the barriers and needs related to physical exercise as experienced by people with ACHD to inform the development of tailored strategies that support and promote increased physical activity.

**Methods:**

Qualitative study using semistructured interviews conducted between March and May 2023. The interview guide was based on the Fear Avoidance Model, Tampa Scale for Kinesiophobia Heart and European Society of Cardiology guidelines on sports cardiology and exercise for cardiovascular diseases. Interviews were coded and thematically analysed to identify specific physical exercise barriers and needs.

**Results:**

Data saturation was reached after interviewing 19 individuals living with ACHD (median age 46 years (range 24–75), 10 women). Thematic analysis identified four main barriers: (1) physical symptoms and negative past experiences, (2) alienation from peers, (3) perceived decline in physical fitness over time and (4) lack of knowledge about personal physical boundaries. Two needs were identified: (1) personalised, disease-specific exercise information and advice and (2) structured support and guidance from healthcare professionals.

**Conclusions:**

People with ACHD face multiple barriers to engaging in physical exercise. There is a clear need for specific, personalised exercise advice from healthcare providers and the development of long-term programmes and interventions to overcome relevant barriers.

**STRENGTHS AND LIMITATIONS OF THIS STUDY**

- Purposeful sampling ensured diversity in backgrounds and health status.
- Inclusion of two academic centres supported broader transferability.
- Predominance of NYHA (New York Heart Association) class I limits representation of severe cases.
- Recruitment from a study addressing physical activity in adults with congenital heart disease may have attracted participants with specific views or experiences.

## Introduction

 The adult congenital heart disease (ACHD) population is growing and ageing, with over 97% of children with congenital heart disease (CHD) now surviving into adulthood.^1 2^ Recent qualitative work has emphasised that adults with ACHD value care that is holistic, psychologically supportive and well coordinated.^3^ Managing CHD is a lifelong process, often involving multiple surgeries and hospitalisations, which, combined with the underlying condition, can limit physical exercise capacity.^1^ Although many people living with ACHD maintain a stable cardiac condition, and the European Society of Cardiology (ESC) guidelines promote safe and regular exercise, approximately three-quarters still express concerns about physical activity.^4 5^ Research in broader cardiovascular populations has indicated that these concerns might be driven by various factors, including kinesiophobia (fear of movement), a lack of personalised guidance and uncertainty about safe exercise practices.^6^,^9^ Given the increased risk for acquired cardiovascular disease, timely preventive strategies, including physical exercise,^10^ are warranted. While Bay *et al*^11^ have provided valuable qualitative insights into barriers and enablers of physical activity in people with ACHD, our study further explored individual needs and preferences within the healthcare context to inform the development of effective, patient-centred exercise interventions. To address this gap, we conducted a qualitative study to explore in depth the physical exercise barriers and needs of people with ACHD.

## Methods

### Research design

We performed a qualitative study using semistructured interviews to explore the perspectives of people living with ACHD on physical exercise. The qualitative study was a nested substudy within the Fear of Movement in Congenital Heart Disease (BACH, Beweegangst bij Congenitale Hartpatienten) study. BACH is a large-scale survey study among adults (>18 years) with ACHD, attending the outpatient clinics of two tertiary care medical centres in the Netherlands, Amsterdam University Medical Center and Erasmus University Medical Center, both specialised in ACHD care. The overall BACH was granted a waiver by the Medical Ethics Review Committee of Erasmus University Medical Center (MEC-2021–0314/A-0001). All individuals provided written informed consent for participation, including the qualitative component. The qualitative study adhered to the Standards for Reporting Qualitative Research guidelines.^12^

### Participants

Individuals were eligible for the qualitative study if they had not been hospitalised or participated in cardiac rehabilitation within the previous 6 months and had consented within the BACH study to be contacted. All invitees received detailed study information and could voluntarily accept or decline. Of the 603 individuals who provided consent, two researchers (YL and MSn) performed purposed sampling based on age, sex and NYHA classification, aiming to capture a range of backgrounds and health statuses.

### Patient and public involvement

A member affiliated with the ACHD patient and public involvement (PPI) group reviewed the interview guide for relevance during the design phase of the study. In addition, the guide was piloted with an individual living with ACHD to assess clarity and identify additional relevant topics.

### Data collection

Patient characteristics (age, sex, educational level, NYHA classification and cardiac rehabilitation participation) were obtained from the BACH study questionnaire, which also included the Tampa Scale for Kinesiophobia Heart (TSK-Heart).^7^ We assessed physical activity levels using one item from the Short Questionnaire to Assess Health-Enhancing Physical Activity,^13^ asking participants how many days per week they engaged in ≥30 min of intensive physical activity.

Qualitative data were collected through semistructured interviews (March to May 2023) online or in private outpatient clinic rooms. We developed the interview guide using the Fear Avoidance Model, the TSK-Heart and the ESC guidelines on sports cardiology.^5 7 14^ The interview guide aimed to cover a comprehensive range of internal (eg, kinesiophobia) and external factors affecting physical exercise. An external review of the interview guide for relevance was conducted by a patient from the ACHD organisation Heart for Research and two cardiologists from the participating medical centres. The interviews started by addressing the patient’s specific type of heart disease, providing context for the subsequent discussion on experiences with physical activity. The type of heart disease was categorised by a cardiologist based on the ESC disease complexity guidelines (mild-moderate-severe).^1^ The focus then shifted to five key themes and their corresponding indicators (table 1). We used non-scripted questions as needed to explore responses in greater depth. The interviews concluded with questions about intervention needs. YL conducted the interviews, with MSn observing a random sample to ensure consistency and reduce observer bias. Sessions were recorded and lasted, on average, 30 min. Key topics were noted afterwards, and iterative data collection enabled the refinement of themes across subsequent interviews. We presented selected patient-level characteristics to support contextual interpretation of participant quotes (online supplemental appendix table 1).

**Table 1:** Topics used in the interview guide

Topic	Indicators
Experiences with healthcare	Feeling supported and acknowledged
	Alignment of cardiac rehabilitation programmes with population needs
Information received or available	Information about heart disease
	Information about physical exercise
Symptoms during physical exercise	Experiencing pain or injury
	Change in physical condition
Fear of movement and cardiac complications	Fear of pain or (re-)injury
	Experiences with physical exercise
	Congenital heart disease perceived as lifelong disability
Passive or dysfunctional coping style	Avoiding physical exercise
	Lack of motivation

### Analysis

We transcribed and coded all interviews using Atlas.ti. Key topics were noted, and iterative data collection allowed for the refinement of topics. YL reviewed all transcripts and notes in line with the first step of Braun and Clarke’s framework for thematic analysis.^15^ Following this, individual codes were generated, organised into subthemes and merged into overarching themes. Two BACH study researchers (MSn and NtH) reviewed the codes and themes to minimise observer bias and support investigator triangulation. Thematic analysis aimed to identify recurring patterns and themes related to the barriers and needs regarding physical exercise. To ensure coherence, topics and indicators (table 1) were mapped to emerging themes, grounding findings in the interview structure. All in-text citations are translated from Dutch to English.

## Results

Data saturation was reached after conducting interviews with 19 individuals living with ACHD as no new themes or insights emerged in the final interviews.

Participants’ median age was 46 years (range 24–75 years); 10 were women, 8 were men and one identified as non-binary or another gender identity. Participants reported engaging in ≥30 min of intensive physical activity on a median of 4 days per week. Median TSK-Heart score was 18, ranging from 13 to 40; 26.3% had TSK-Heart scores indicative of kinesiophobia (table 2).

**Table 2:** Participant characteristics

Characteristics (n=19)	Measurement
Age in years, median (min; max)	46 (24; 75)
Sex, n (%)	
Men	8 (42.1)
Women	10 (52.6)
Non-binary	1 (5.3)
Level of educational attainment*, n (%)	
Low	2 (10.5)
Intermediate	8 (42.1)
High	9 (47.4)
Classification of congenital heart disease complexity†, n (%)	
Mild	5 (26.3)
Moderate	11 (57.9)
Severe	3 (15.8)
NYHA classification‡, n (%)	
I	13 (68.4)
II	5 (26.3)
III	1 (5.3)
TSK-score§, median (min; max), n (%)	18 (13; 40)
Low score (13–27)	14 (73.7)
High score (28–52)	5 (26.3)
Prior participation in cardiac rehabilitation, n (%)	9 (47.4)
Number of days per week with ≥30 min intensive physical activity, median (min; max)	4 (0; 7)

* Highest educational attainment: low=primary education, secondary education (VMBO, MBO-1); intermediate=HAVO, VWO, intermediate and specialist vocational training (MBO-2/MBO-3/MBO-4); high=university of applied sciences (HBO) and research university education.^16^

† ESC classification of congenital heart disease based on complexity, guiding clinical management and risk assessment.^1^

‡ NYHA classification: I=no limitation; II=slight limitation; III=marked limitation; IV=symptoms of heart failure.^21^

§ TSK scores.^7^

ESC, European Society of Cardiology; TSK, Tampa Scale for Kinesiophobia.

### Fear of movement

Many participants stated they did not fear physical activity and exercised regularly. They recognised the health benefits of physical exercise, including ‘improved heart fitness’, maintaining a healthy weight and aiding in recovery after surgery and in healthy ageing. Even those with higher levels of kinesiophobia reported that the satisfaction of exercising outweighed their fear, motivating them to continue to exercise. Although relatives reported greater concerns than participants themselves about future cardiac incidents, this did not prevent interviewees from participating in physical exercise.

It is not that I don't dare to get on a treadmill because I fear I will get too tired or suffer or something serious will happen. I do not have that. (P11)

#### Barriers and needs

**Figure 1:**
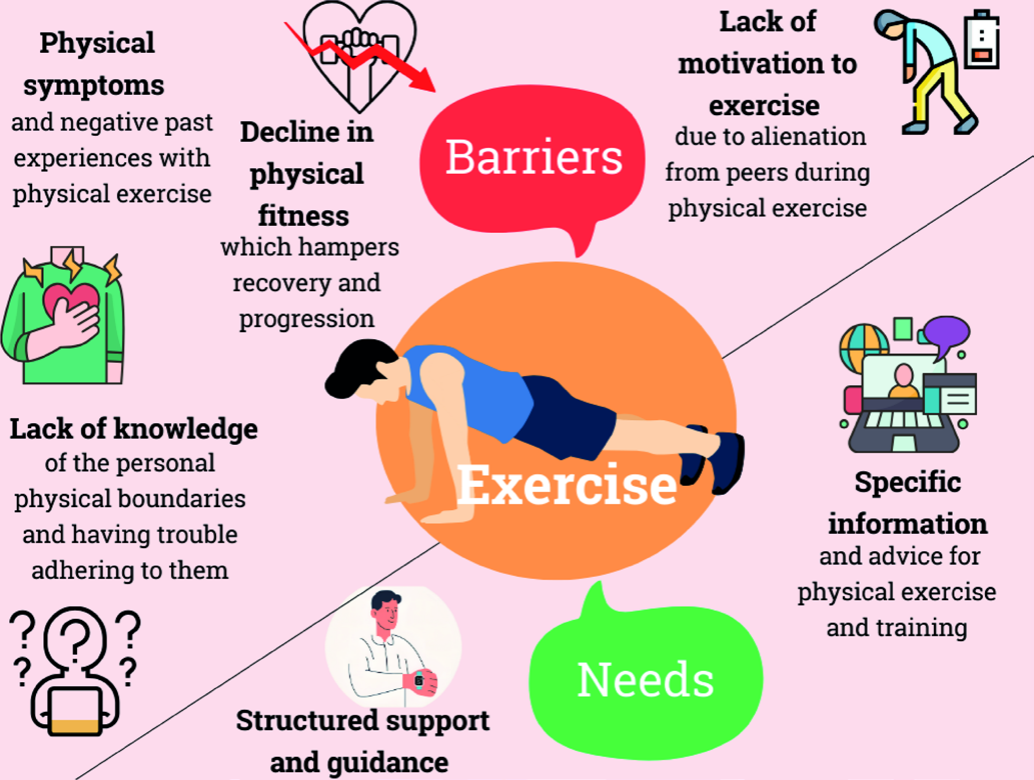
Overview of barriers and needs of people with ACHD. ACHD, adults with congenital heart disease.

Thematic analysis identified themes highlighting four barriers and two needs for people with ACHD. The barriers included (1) physical symptoms and negative past experiences, (2) alienation from peers, (3) perceived decline in physical fitness over time and (4) lack of knowledge about personal physical boundaries. Two needs were identified: (1) personalised, disease-specific exercise information and advice and (2) structured support and guidance from a healthcare professional. An overview of the barriers and needs is provided in figure 1.

### Barriers to physical exercise

#### Physical symptoms and negative experiences with exercise

Interviewees reported experiencing physical symptoms during physical exercise, such as cardiac arrhythmias and chest pain, which led them to approach physical exercise with caution. These individuals reported monitoring their heart rate, avoiding exercising alone or in isolated areas and refraining from pushing themselves to their maximum capacity to minimise potential risks. Some participants shared negative experiences, such as fainting and cardiac arrest, which diminished their confidence in their physical abilities. This was particularly evident with higher-intensity sports, such as running, soccer and vigorous hiking, resulting in reduced confidence and a tendency to avoid these types of exercises.

#### Alienation from peers

Interviewees reported alienation from their peers during physical exercise, which contributed to a negative view of physical exercise. They perceived themselves as having a lower exercise capacity than others of their age and often felt like outsiders. For some, this feeling originated from childhood challenges with team sports, which discouraged them from exercising as adults. Additionally, they observed that building and maintaining fitness required greater motivation and effort than for those without ACHD, making it challenging to find sports they could enjoy and to prioritise exercise. Nevertheless, some reported finding motivation in the social aspect of sports, particularly when exercising with individuals at similar intensity levels, where mutual encouragement helped reduce barriers to participation.

It does take a bit more energy, at least I have to train as hard as someone going for the marathon, for the five kilometres. (P11)

#### Perceived decline in physical fitness over time

Interviewees reported a gradual decline in physical fitness over time, making regular exercise increasingly challenging. They attributed this decline to their heart condition, perceiving that progressive reduction in cardiac function increasingly limited their endurance and impeded their ability to improve their fitness level. Approximately half of the interviewees perceived their ACHD as a lifelong limitation to healthy ageing. The sense of declining physical fitness was more prevalent among older individuals and those with moderate to severe heart disease.

It also really has to do with getting older in a healthy way and I feel hampered in that. When I see what people of my age around me can do, people stay fitter all the time, and I notice that I am lagging others in that respect. (P15)

#### Lack of knowledge about personal physical boundaries

Participants reported halting exercise when experiencing symptoms like palpitations or chest discomfort, often feeling uncertain about their boundaries or the appropriate type or intensity of exercise. They rarely consulted their physician about this and reported receiving only general exercise advice during hospital visits. This lack of knowledge made some hesitant to exert full effort during exercise. Others admitted to ignoring symptoms in an attempt to ‘prove’ their health and maximise their performance in sports.

In primary school or high school when you had those beep tests, I was often the only one still running. Then there is no yardstick to say, ‘maybe I should stop now’? (P10)

### Healthcare needs

#### Personalised disease-specific exercise information and advice

Interviewees emphasised the importance of receiving personalised exercise information, including recommendations on suitable types of sports, appropriate intensity levels and clear physical boundaries. They preferred this advice to come from cardiologists and cardiac rehabilitation professionals. Many participants noted that they currently received only general advice and lacked sufficient knowledge to develop effective exercise routines. Furthermore, physical exercise was seldom discussed during hospital visits, and limited contact with healthcare providers left many of their questions unanswered. Although most participants underwent (cardiopulmonary) exercise tests, they were often unaware of the results and potential implications for exercise.

It seems to me that a cardiologist is mostly concerned with: How am I going to make sure you leave the door fit or in good health again, but never thinks: How am I going to help someone move forward again? (P4)

#### Structured support and guidance from healthcare professionals

Participants noted that cardiac rehabilitation often failed to align with their actual need for support, as the actual phase of building up their training comes later in the recovery process. They expressed a strong desire for more comprehensive and extended guidance beyond the standard 6- to 12-week cardiac rehabilitation programmes. The inclusion of mixed-age groups and varying fitness levels in these programmes was described as particularly discouraging. In the absence of personalised support and guidance, many participants relied solely on their own insights to stay active. Some turned to external resources for additional support, such as personal trainers, smartwatch monitoring and organisations like the Heart Foundation. While some participants found these sources helpful, others lacked access to or awareness of them, leaving them without adequate guidance for recovery and achieving their physical activity goals.

Interviewees suggested, in addition to exercise apps or monitoring, organising dedicated gatherings where tailored exercise recommendations would be combined with professional support. This idea reflected two distinct needs: peer support and individualised guidance. Middle-aged participants (40–65 years), who were particularly affected by decline in physical fitness, strongly emphasised this need. These insights underline the importance of designing support programmes that balance social connection with tailored advice to effectively meet patient needs. Additionally, participants reported that Cardiopulmonary Exercise Testing (CPET) was rarely conducted. Even when it was performed, they often did not receive feedback on their personal exercise zones or physical capacity. The absence of individualised recommendations based on CPET results was perceived as a missed opportunity.

I already felt so good that I thought, is this all there is? It (cardiac rehabilitation) was intended for a different target group, which I did not feel belonged to. (P6)

## Discussion

This qualitative study identified four central physical exercise barriers and two key needs in individuals living with ACHD. Barriers comprised exercise limitation due to physical symptoms and negative past experiences, alienation from peers, a decline in physical fitness over time and a lack of knowledge about personal physical boundaries. Key needs constituted the availability of personalised, disease-specific exercise guidance and the need for structured support and guidance from healthcare professionals. The findings offer clear targets for the development of interventions aiming to improve levels of physical activity.

Based on existing literature and clinical observations, we considered the possibility that kinesiophobia, that is, fear of movement, might constitute an important barrier to physical exercise.^7 9^ However, most interviewees reported little or negligible levels of kinesiophobia, and even when present, kinesiophobia appeared to have minimal effect on exercise participation. Participants did, however, voice concerns about exercising safely and did indicate feeling uncertain about their physical boundaries. These findings underscore the importance of addressing the lack of knowledge regarding appropriate exercise intensity and highlight that measuring kinesiophobia might be less important to address physical exercise barriers.

In line with our outcomes, studies have identified knowledge gaps, uncertainty and motivational factors as barriers to exercise in cardiovascular patients.^6 8 11^ Our findings support earlier work on motivational and contextual factors, including early-life encouragement and environmental support.^11^ We further add depth to existing research by showing the need for individualised, condition-specific exercise advice and structured guidance from healthcare professionals. The identified barriers and needs in our study might be interconnected. A lack of knowledge about physical boundaries can lead to negative exercise experiences, exacerbating feelings of alienation and diminishing motivation to remain active. Similarly, a decline in physical fitness over time may reinforce negative perceptions of exercise and increase the demand for tailored guidance and support.

Individuals with ACHD have reported that conventional rehabilitation programmes lack customisation and ongoing support, emphasising the need for more personalised and sustained interventions. This aligns with findings by Sheng *et al*,^16^ who showed that only 47% of people with ACHD completed the recommended cardiac rehabilitation sessions, highlighting limited engagement. While current cardiac rehabilitation programmes could provide a foundation for implementing more tailored approaches, significant adaptations are required to meet the unique needs of people with ACHD. Many are less frequently referred to traditional rehabilitation due to restrictive eligibility criteria or having undergone surgery at an early age. Additionally, existing programmes often fail to provide long-term, individualised support. Developing rehabilitation protocols that account for the diverse types and severities of congenital heart conditions could help bridge these gaps and improve patient engagement.

Our findings indicate that participants often did not receive feedback from CPET, which they perceived as a missed opportunity for tailored exercise guidance. This finding aligns with the 2020 ESC guidelines, which emphasise tailoring exercise to individual risk profiles.^5^ Integrating CPET with personalised feedback could bridge this gap, enhancing patient outcomes. Additionally, eHealth platforms may offer a promising avenue for delivering flexible, personalised and remote exercise guidance, extending support beyond traditional rehabilitation settings.^17 18^

### Strengths and limitations

This qualitative study captures the in-depth experiences of people with and identifies specific needs to guide the development of future exercise support programmes.

Including a varied population from two major University Medical Centres enhances the transferability of our findings by capturing a broader range of patient experiences, needs and barriers across two geographical regions. Some aspects of our study warrant consideration. We used purposed sampling to capture a range of backgrounds and health statuses. However, our sample was drawn from the BACH study, which focused on physical exercise and sports in people with ACHD. This may have introduced responder bias, skewing participation towards either highly active individuals or those facing severe barriers.

While the sample included a range of NYHA classifications, the majority of participants had mild disease severity, limiting representation of those with more complex physical restrictions. This reflects the broader demographic profile of the ACHD population, in which many individuals present with relatively mild symptoms in adulthood. Lastly, recall bias may have influenced the findings, as participants reflecting on their exercise experiences could have memories shaped by their current health status or recent experience.^19 20^

In line with the ESC Working Group’s call for timely preventive strategies in ACHD,^9^ our findings emphasise the need for structured, personalised exercise guidance to address patient-specific barriers and promote safe physical activity engagement. Such guidance could help overcome key obstacles, including alienation from peers, negative exercise experiences and declining physical fitness, ultimately empowering participation more confidently in physical activity and sports.

Further research and education on safe exercise intensity are essential, particularly for those uncertain about their capabilities. Additionally, future studies should focus on identifying and evaluating the most effective methods for delivering personalised exercise guidance. To strengthen these clinical implications, it is also important to assess the effectiveness of tailored interventions in improving exercise participation and long-term adherence among people with ACHD.

In conclusion, this study highlights the multiple barriers individuals living with ACHD face in engaging in physical exercise. While kinesiophobia was reported by few, many expressed caution due to negative symptoms, past experiences and uncertainty about their physical limits. There is a clear need for tailored, professional exercise guidance and long-term programmes to address these challenges. Such interventions could enhance confidence in physical activity, improving quality of life and reducing health risks associated with inactivity.

## Supplementary material

10.1136/bmjopen-2025-102090 online supplemental file 1

## Data Availability

Data sharing not applicable as no datasets generated and/or analysed for this study.
