# Supplementary material for: Physical exercise barriers and needs in adults with congenital heart disease: a qualitative study
Source: BMJ Open. 2025 Jul 5;15(7):e102090. doi: 10.1136/bmjopen-2025-102090 (PMC12228472; doi:10.1136/bmjopen-2025-102090)
Supplement: online supplemental file 1 [file bmjopen-15-7-s001.docx]

Supplementary Table. Individual participant characteristics.

| Patient | Age (January 1^st^ 2023) | Sex | Classification of congenital heart disease complexity^a^ | Number of days per week with =>30 minutes intensive physical activity, median (min; max) |
| --- | --- | --- | --- | --- |
| P1 | Late 20s | Female | Mild | 4 |
| P2 | Mid 70s | Female | Mild | 1 |
| P3 | Late 40s | Female | Mild | 7 |
| P4 | Early 40s | Male | Moderate | 5 |
| P5 | Mid 50s | Male | Moderate | 2 |
| P6 | Late 50s | Female | Mild | 3 |
| P7 | Mid 40s | Male | Moderate | 5 |
| P8 | Mid 30s | Female | Mild | 4 |
| P9 | Mid 20s | Female | Severe | 7 |
| P10 | Early 30s | Male | Moderate | 7 |
| P11 | Late 30s | Female | Moderate | 2 |
| P12 | Early 30s | Male | Moderate | 6 |
| P13 | Early 50s | Male | Severe | 0 |
| P14 | Mid 20s | Other | Moderate | 6 |
| P15 | Late 50s | Female | Moderate | 3 |
| P16 | Late 40s | Female | Severe | 0 |
| P17 | Early 30s | Male | Moderate | 7 |
| P18 | Late 40s | Male | Moderate | 5 |
| P19 | 67 | Female | Moderate | 1 |

^a^ESC classification of congenital heart disease based on complexity, guiding clinical management and risk assessment (1)

^b^NYHA-classification: I = no limitation; II = slight limitation; III = marked limitation; IV = symptoms of heart failure (16)
