# Supplementary material for: Physical exercise barriers and needs in adults with congenital heart disease: a qualitative study
Source: BMJ Open. 2025 Jul 5;15(7):e102090. doi: 10.1136/bmjopen-2025-102090 (PMC12228472; doi:10.1136/bmjopen-2025-102090)
Supplement: Supplementary file 2 [file bmjopen-2025-102090.reviewer_comments.pdf]

PEER REVIEW HISTORY

BMJ Open publishes all reviews undertaken for accepted manuscripts. Reviewers are asked to complete a checklist review form and are provided with free text boxes to elaborate on their assessment. These free text comments are reproduced below.

ARTICLE DETAILS

Title (Provisional)

Physical Exercise Barriers and Needs of Adult Congenital Heart Disease Patients. A qualitative study.

Authors

Langeveld, Yara F.; ter Hoeve, Nienke; van den Bosch, Annemien; Robbers-Visser, Danielle; Kauling, Robert M; van Groen, Jasmijn C.; Sunamura, M.; Jorstad, Harald; Snaterse, Marjolein

---

VERSION 1 - REVIEW

**Reviewer**	**1**
**Name**	**Bay, Annika**
**Affiliation**	**Umeå Universitet Medicinska fakulteten, Nursing**
**Date**	**15-Apr-2025**
**COI**	**None**

Physical Exercise Barriers and Needs of Adult Congenital Heart Disease Patients. A qualitative study.

Overall, the study presents very interesting and important findings. There is a need of more qualitative research in this area.

Introduction

Generally well described introduction, however, I lack previous qualitative research in the field. See for example Bay et al 2021

The aim differs from the aim in the abstract.

Methods

You write that you made a purposive sample based on age, gender and NYHA classification, aiming to capture a range of backgrounds and health statuses. Were the informants allowed to enroll in the study themselves and if so, how was this done? Were they all asked, or did you select those you thought were suitable? Please, elaborate on how you did.

Results

Your findings are consistent with previous research in this area from other countries, which strengthens your study. However, you have different names for your themes in the abstract, results and figure. Are they really themes or are they sub-themes you describe, the text may need to be processed more thoroughly to eventually result in themes that are a little more comprehensive.

It is an advantage if you report which participant you are quoting, it is sufficient to write p1, p2, etc.

Discussion

Writing the discussion is usually the most difficult part, here it is important that you think about what are your most important findings and why? In general, the discussion could benefit from being developed and, above all, discussed more in relation to what is described in previous qualitative research in the field.

Page 15, line 28; "This qualitative study identified four central physical exercise barriers and two key needs in" - you describe five barriers in the result, what is correct?

Page 15, line 50; "We hypothesised that kinesiophobia, i.e. fear of movement, might constitute an important barrier to physical exercise" - Hypotheses do not occur in qualitative research, please rephrase.

Page 16, line 16; "In line with previous research" - what previous research? The discussion would benefit from being discussed against previous research.

**Reviewer**	**2**
**Name**	**Maiorana, Andrew**
**Affiliation**	**Curtin University, School of Physiotherapy and Exercise Science**
**Date**	**18-Apr-2025**
**COI**	**None**

General Comments:

Overall, a well-designed, sound application of qualitative research methodology to an important topic.

Several statements are made in a way that implies they are evidence-based but lack citations, please ensure all such statements are referenced.

Specific comments

The term "ACHD patients" is used throughout. It would be better to describe the cohort as "people with ACHD" so as not to define the person by their condition.

Page 4, reference line 47 – “Facilitators” are mentioned but not described elsewhere in the study, other than a single mention in passing in the Discussion (page 17, line 55). This is not a suggestion to remove reference to facilitators but rather include information about facilitators. See comment below regarding *“Fear of exercise”* as an example.

Page 5, ref lines 21-23. BACH was granted a waiver by the Medical Ethics Review Committee of Erasmus University Medical Centre but the qualitative study collected written informed consent, was there an ethics approval for the qualitative study?

Page 6, ref lines 33-36 – re. “the interviews started by addressing the patient’s specific type of heart disease, categorized by a cardiologist based on the ESC disease complexity guidelines…” – It’s not clear what “addressing” refers to in this context.

Page 8, ref line 33 – How was data saturation determined?

Table 2 – Patient characteristics. For sex, one participant is listed as “Other”. It would be more inclusive to name how they identify, for example ‘non-binary”.

Table 2 – Patient characteristics. Including details of the aetiology of the participant cohort would be useful.

Page 8, ref line 43 – How was data saturation determined?

Page 11 – “Fear of Exercise” – This section doesn’t seem appropriate as a “*Barrier to exercise”* given that the feedback from patients is positive about exercise. It would be more appropriate framed as a *Facilitator* of exercise.

Page 16, ref lines 16-21 re. “In line with previous research, which has identified knowledge gaps and hesitancy toward participation as barriers to exercise in cardiovascular patients…” and lines 25-30, “A lack of knowledge about physical boundaries can lead to negative exercise experiences, exacerbating feelings of alienation and diminishing motivation to remain active” Please provided citations for the previous research being referred to or justifying these claims.

Page 16, ref line 37 _ “ACHD patients have reported that conventional rehabilitation programs lack customization and ongoing support”. “Have” is redundant in this sentence if the current study is being referred to. The authors go on to state that people with ACHD have limited engagement, which aligns with the findings of Sheng et al. (16), who reported that only 47% of ACHD patients complete the recommended cardiac rehabilitation sessions. How does this compare with the completion rate amongst people with acquired CVD?

Page 17, ref lines 42-44 re: “Aspects of our study warrant consideration”. This section describes limitations of the study and it would be best to describe these “aspects” as such. Acknowledging limitations is not necessarily a negative reflection on the study but rather puts the findings in the context that they have been acquired.

Page 18, ref lines 8-11. Re. “patients in NYHA classes I and II are over-represented in the overall population of ACHD” seems an unusual way to describe the fact that many peoples with congenital cardiac abnormalities have relatively modest symptoms (just stating the latter would be sufficient to illustrate this point).

Standards for Reporting Qualitative Research (SRQR) - Qualitative approach and research paradigm – It’s unclear what “reasonably yes” means?

VERSION 1 - AUTHOR RESPONSE

Reviewer 1 – Dr. Annika Bay

General comment

Overall, the study presents very interesting and important findings. There is a need of more qualitative research in this area.

We thank the reviewer for this encouraging assessment and recognition of the need for more qualitative work in this field.

Introduction

I lack previous qualitative research in the field. See for example Bay et al 2021.

Response: We agree that previous qualitative research is relevant to contextualize our findings. We have now incorporated Bay et al. (2021) and Wray et al. (2024) to acknowledge the existing qualitative literature. In the introduction, we refer to Bay et al. to illustrate how motivational and contextual factors influence physical activity behaviour in ACHD patients. In the discussion, we further relate our findings to theirs and emphasize how our study builds on this by highlighting patients' specific needs for tailored and structured guidance.

Change:

Introduction, p.4

Recent qualitative work has emphasized that adults with ACHD value care that is holistic, psychologically supportive, and well-coordinated (Wray et al., 2024).

Introduction, p.4

While Bay et al. (11) have provided valuable qualitative insights into barriers and enablers of physical activity in people with ACHD, our study further explored individual needs and preferences within the healthcare context to inform the development of effective, patient-centred exercise interventions

Discussion, p. 16

In line with our outcomes, ~~previous research, which has~~ studies have identified knowledge gaps, uncertainty, and motivational factors ~~and hesitancy toward participation~~ as barriers to exercise in cardiovascular patients (6,8,11). Our findings ~~further~~ support earlier work ~~emphasise~~ on motivational and contextual factors, including early-life encouragement and environmental support al. (11). We further add depth to existing research by showing the need for individualised, condition-specific exercise advice and structured guidance from healthcare professionals.

The aim differs from the aim in the abstract.

Response: We have revised the objective in the abstract to align with the aim in the introduction
Change: Abstract, objective, p.2
However, many individuals with ACHD ~~patients~~, remain insufficiently physically active. ~~Identifying physical exercise barriers and needs experienced by ACHD patients is required to develop effective, tailored interventions to increase exercise levels.~~ This study explored the barriers and needs related to physical exercise as experienced by people with ACHD, to inform the development of tailored strategies that support and promote increased physical activity.

Introduction p. 4
To address this gap, we conducted a qualitative study to explore in depth the physical exercise barriers ~~facilitators~~ and needs of people with ACHD ~~patients~~ ~~in depth~~.

Methods
Were the informants allowed to enroll in the study themselves and if so, how was this done? Please elaborate.
Response: As described in the Methods section under 'Research design' and 'Participants', the qualitative study was a nested substudy within the Fear of Movement in Congenital Heart Disease (BACH) study and all interviewees had provided prior consent within the BACH study to be approached for follow-up qualitative research. The overall BACH study is self-enrolled. Participants for our qualitative study were subsequently purposively selected by the research team, based on predefined characteristics. There was no self-enrolment outside this process. To avoid ambiguity, we have clarified this point further in the revised manuscript.
Change: Methods, Participants section, p. 5
~~Patients~~ Individuals were eligible for the qualitative study if they had not been hospitalised or participated in cardiac rehabilitation within the previous six months and had ~~previously provided~~ consented within the BACH study to be contacted. ~~approached for the qualitative study~~. All invitees received detailed study information and could voluntarily accept or decline.

Results
You have different names for your themes in the abstract, results, and figure. Are they really themes or sub-themes?
Response: We thank the reviewer for pointing this out.
To clarify:

1. The topic list in Table 1 reflects the structure of the interview guide, which was developed a priori to broadly explore participants' experiences and perceptions of physical activity and healthcare in the context of ACHD.

2. The themes presented in the results section, such as *"declining physical fitness"* or *"alienation from peers,"* were inductively derived through thematic analysis of the interview transcripts. These reflect patterns and meanings that emerged from the data.
3. The figure (and abstract) summarizes these final analytical themes, sometimes grouping them under higher-order conceptual categories.

We have now clarified in the manuscript that Table 1 reflects the structure of the interview guide and not the final analytical themes, to avoid potential confusion. To improve clarity and consistency, we have revised the manuscript to ensure that the barriers and needs identified through thematic analysis are formulated uniformly across the abstract, results and discussion.
The following themes are now used consistently throughout the manuscript:
Barriers:

1. Physical symptoms and negative past experiences with exercise
2. Alienation from peers
3. Perceived decline in physical fitness over time
4. Lack of knowledge about personal physical boundaries

Needs:

1. Personalised, disease-specific exercise information and advice
2. Structured support and guidance from healthcare professionals

Change: Methods, Analysis, p. 7
~~The~~ Thematic analysis aimed to identify recurring patterns and themes related to the barriers and needs regarding physical exercise. ~~This rigorous process allowed for the development of a structured understanding of the data.~~ To ensure coherence, topics and indicators (Table 1) were mapped to emerging themes, grounding findings in the interview structure.

Change: Throughout manuscript. Abstract p. 2 , Results p. 10, section headings from p. 11 onwards, Results from p.11, Figure (separate), and Discussion p. 15.
Results, p. 11
Thematic analysis identified themes highlighting four barriers and two needs for people with ACHD ~~patients~~. The barriers included 1) physical symptoms ~~experienced during exercise~~ and negative past experiences, 2) alienation from peers, 3) perceived decline in physical fitness over time, and 4) lack of knowledge about personal physical boundaries. Two needs were identified: 1) personalised, disease-specific exercise information and advice, and 2) structured support and guidance from healthcare professional. An overview of the barriers and needs is provided in Figure 1.

It is an advantage if you report which participant you are quoting (p1, p2, etc.).

Response: We thank the reviewer for this helpful suggestion. We agree that attributing quotes to individual participants (e.g., P1, P2) can enhance transparency and contextual understanding. In response, we have now added participant identifiers to the quotes in the results section. Additionally, we have included a supplementary table in the appendix presenting key participant-level characteristics (e.g., age, sex, ESC classification of heart disease, physical activity level).
This approach provides readers with contextual background for each quote while safeguarding anonymity, especially given the relatively small and specific patient population. It also aligns with qualitative research standards and ethical reporting practices, including the COREQ checklist, by balancing transparency with protection of participant identity.

Change: Methods, data collection, p.5 We presented selected patient-level characteristics to support contextual interpretation of participant quotes (Appendix Table 1).

Discussion
Discussion could benefit from more integration with previous qualitative research in the field.
Response: We agree with the reviewer that the discussion benefits from further integration with previous qualitative research. As noted in our response to the first comment, we have now referenced and incorporated findings from Bay et al. (2021) among others and aligned our interpretation more explicitly with earlier work.
Change:
Discussion, p. 16
In line with our outcomes, ~~previous research, which has~~ studies have identified knowledge gaps, uncertainty, and motivational factors ~~and hesitancy toward participation~~ as barriers to exercise in cardiovascular patients (6,8,11). Our findings ~~further~~ support earlier work ~~emphasise~~ on motivational and contextual factors, including early-life encouragement and environmental support al. (11). We further add depth to existing research by showing the need for individualised, condition-specific exercise advice and structured guidance from healthcare professionals.

Page 15, line 28; you describe five barriers in the results, what is correct?
Response: Thank you for your careful observation. We agree that the section on "fear of movement" (kinesiophobia) was incorrectly placed under "Barriers to physical exercise." As also addressed in response to Reviewer 2, we have moved this content to the preceding section, where it is now presented as part of the participants' broader experiences with physical activity, rather than as a barrier.
As a result of this change, the results section now describes four barriers and two needs, which we have clarified in both the text and structure of the manuscript.
Change:

Replacement in Results. Section title 'fear of movement' removed from 'barriers to physical exercise'. p. 10

Page 15, line 50; Hypotheses do not occur in qualitative research, please rephrase.
Response: We agree with the reviewer that hypotheses are not typically formulated in qualitative research. Our initial interest in kinesiophobia as a potential barrier was instead informed by theoretical models (e.g., the fear-avoidance model, Health Belief Model), prior quantitative studies, and clinical observations suggesting that fear of movement may reduce physical activity.
Change: Discussion, p. 15
~~We hypothesised~~ Based on existing literature and clinical observations, we considered the possibility that kinesiophobia, i.e. fear of movement, might constitute an important barrier to physical exercise (7,9). However, most interviewees reported little or negligible levels of kinesiophobia, and even when present, kinesiophobia appeared to have minimal effect on exercise participation.

"In line with previous research" - what previous research?
Response: We thank the reviewer for this observation and have added citations to relevant literature.
Change: Discussion, p. 16
In line with our outcomes, ~~previous research, which has~~ studies have identified knowledge gaps, uncertainty, and motivational factors ~~and hesitancy toward participation~~ as barriers to exercise in cardiovascular patients (6,8, 11). While Bay et al. (11) ~~our findings further~~ emphasise motivational and contextual factors, including early-life encouragement and environmental support, our study add depth to existing qualitative research by showing the need for individualised, condition-specific exercise advice and structured guidance from healthcare professionals.

Reviewer 2 – Dr. Andrew Maiorana
General comment
Overall, a well-designed, sound application of qualitative research methodology to an important topic.
Thank you for your kind assessment.

Several statements are made in a way that implies they are evidence-based but lack citations, please ensure all such statements are referenced.
We have carefully reviewed the manuscript and added references where evidence-based claims were made without citation. In cases where the statements were based on our own

interpretation or reflect commonly accepted knowledge, we clarified the phrasing accordingly.

Specific comments

Use "people with ACHD" instead of "ACHD patients".

Response: Thank you for your valuable comment regarding person-first language. We agree with the importance of using respectful and person-centered terminology, and have revised the manuscript accordingly. This includes consistent changes throughout the text as well as an adjustment of the title.

Change: Throughout manuscript and title:

Physical Exercise Barriers and Needs in ~~of~~ Adults with Congenital Heart Disease ~~Patients~~. A qualitative study

Page 4, ref line 47 – "Facilitators" are mentioned but not described elsewhere in the study.

Response: Thank you for this helpful comment. We agree that we did not explicitly categorize 'facilitators' as a separate theme. In our analysis, elements that could be considered facilitator, such as guidance, clear information, and support, were captured under participants' *needs* for physical activity. We have now clarified this in the manuscript to avoid confusion, and adjusted the wording accordingly.

Change: Introduction, p.4

To address this gap, we conducted a qualitative study to explore in depth the physical exercise barriers ~~facilitators~~ and needs of people with ACHD ~~patients~~ ~~in depth~~.

Discussion, p.18

Despite this, the identified barriers and ~~facilitators~~ needs remain highly relevant, offering insights into both motivations and challenges in exercise participation

Page 5, ethics – was there separate approval for the qualitative study?

Response: The qualitative study was part of the broader Fear of Movement in Congenital Heart Disease (BACH) study and was explicitly included in the original submission to the Medical Ethics Review Committee of Erasmus University Medical Centre. The committee granted a waiver for the entire BACH study (MEC-2021-0314/A-0001), which covered the broader research framework, including the qualitative component.

Change: Methods, research design, p. 5

The qualitative study was a nested sub-study within the Fear of Movement in Congenital Heart Disease (BACH) study. ~~In short,~~ BACH is a large-scale survey study among adults (≥18 years) with ACHD ~~patients~~, attending the outpatient clinics of two tertiary care medical centres in the Netherlands, Amsterdam University Medical Center and Erasmus University Medical Center, both specialised in ACHD care. The overall BACH was granted a waiver by the Medical Ethics Review Committee of Erasmus University Medical Center (MEC-2021-0314/A-0001).

Page 6 – “addressing the patient’s specific type of heart disease” – clarify “addressing”
Response: Thank you for your comment. We have rephrased this sentence to clarify that the interviews began with an open conversation about the patient’s specific heart condition, which served as contextual grounding for the rest of the discussion.
Change: Methods, Data collection, p. 6
The interviews started by addressing the patient’s specific type of heart disease, providing context for the subsequent discussion on experiences with physical activity.

Page 8 – How was data saturation determined?
Response: We have added an explanation of how data saturation was evaluated, based on recurrence of themes and no new insights emerging.
Change: Results, p. 8
Data saturation was reached after conducting interviews with 19 individuals living with ACHD as no new themes or insights emerged in the final interviews.

Table 2 – one participant is listed as “Other” for sex; consider “non-binary”
Response: We have revised the label to ‘non-binary’ to reflect inclusive and respectful language.
Change: Results, Table 2. p.9
Non-binary

Include aetiology of heart disease in patient characteristics.
Response: Thank you for your suggestion. While we recognize the potential value of including the aetiology of heart disease, we chose to report disease complexity using the ESC classification (mild–moderate–severe) as a clinically relevant and non-identifiable way to describe our diverse sample. Given the small sample size and wide range of diagnoses, reporting individual aetiologies could compromise participant anonymity. Moreover, in many adult congenital heart disease (ACHD) patients, the original aetiology may no longer fully capture the clinical picture. Multiple surgical interventions and evolving complications mean that disease trajectories can vary substantially, and comparing aetiologies alone may not adequately reflect this complexity.
This approach is consistent with other qualitative research in congenital heart disease populations (e.g., Nemes et al. Growing Up With Congenital Heart Disease: A Qualitative Research Study of Parents’ and Clinicians’ Experiences and Perspectives. CJC Pediatr Congenit Heart Dis. 2025;4(1):22–33).

“Fear of exercise” section seems more like a facilitator.
Response: We agree that this section was placed incorrectly and does not belong under "Barriers to physical exercise." We have moved the content on fear of movement

(kinesiophobia) to the section above, where it is now described as part of the participants' experiences with physical activity.
Change: Replacement in Results. Section title 'fear of movement' removed from 'barriers to physical exercise'. p. 10

Provide references for previous research mentioned on Page 16.
Response: We thank the reviewer for this observation and have added citations to relevant literature. In addition to broader cardiovascular studies (e.g., Budts et al., Bäck et al.), we now cite Bay et al. (2021), a qualitative study among ACHD patients reporting on similar barriers. Our findings build on this work by identifying less explored aspects such as peer-related alienation and the expressed need for personalised, long-term support. We have updated the discussion accordingly.
Change: Discussion, p. 16. Added citations.

"Have reported" – redundancy.
Response: Sentence was revised to correct redundancy.
Change: Discussion, p. 16
This aligns with findings by Sheng et al. (16), who showed ~~reported~~ that only 47% of people with ACHD ~~patients~~ completed the recommended cardiac rehabilitation sessions, highlighting limited engagement

Limitations section: name them as such.
Response: We changed phrasing to explicitly name this section as Strengths and limitations and restructured accordingly.
Change: Discussion, p.17
Strengths and limitations

Rephrase: "people in NYHA I and II are over-represented…"
Response: We have revised the sentence to improve clarity and to avoid any unintended implications associated with the term "over-represented." The revised phrasing better reflects our intended meaning in a more neutral and respectful manner.
Change: Discussion, p. 18
While the sample included a range of NYHA classifications, the majority of participants had mild disease severity, limiting representation of those with more complex physical restrictions.

Clarify "reasonably yes" in SRQR table.
Response: We have revised the relevant item in the SRQR checklist (p. 23) to clearly state the research paradigm. We now specify that the study was conducted from an interpretivist paradigm, using semi-structured interviews and reflexive thematic analysis to explore participants' subjective experiences. This methodological approach aligns with the study aim

and supports the generation of in-depth insights into the participants' perspectives and needs.
Change: SRQR checklist, p.23
Yes

VERSION 2 - REVIEW

**Reviewer**	**1**
**Name**	**Bay, Annika**
**Affiliation**	**Umeå Universitet Medicinska fakulteten, Nursing**
**Date**	**13-Jun-2025**
**COI**	

Thank you for your clarifying answers, I have no further comments. Your work contributes to a better understanding and knowledge of the importance of support and individualised advice regarding physical activity in this group.
